# Mating type specific transcriptomic response to sex inducing pheromone in the pennate diatom *Seminavis robusta*

**DOI:** 10.1038/s41396-020-00797-7

**Published:** 2020-10-07

**Authors:** Gust Bilcke, Koen Van den Berge, Sam De Decker, Eli Bonneure, Nicole Poulsen, Petra Bulankova, Cristina Maria Osuna-Cruz, Jack Dickenson, Koen Sabbe, Georg Pohnert, Klaas Vandepoele, Sven Mangelinckx, Lieven Clement, Lieven De Veylder, Wim Vyverman

**Affiliations:** 1 Protistology and Aquatic Ecology, Department of Biology, Ghent University, 9000 Ghent, Belgium; 2 Department of Plant Biotechnology and Bioinformatics, Ghent University, 9052 Ghent, Belgium; 3 VIB Center for Plant Systems Biology, 9052 Ghent, Belgium; 4 Department of Applied Mathematics, Computer Science and Statistics, Ghent University, 9000 Ghent, Belgium; 5 Department of Statistics, University of California, Berkeley, Berkeley, CA USA; 6 SynBioC, Department of Green Chemistry and Technology, Ghent University, Coupure Links 653, 9000 Ghent, Belgium; 7 B CUBE Center for Molecular Bioengineering, Technical University of Dresden, Tatzberg 41, 01307 Dresden, Germany; 8 Bioinformatics Institute Ghent, Ghent University, Technologiepark 71, 9052 Ghent, Belgium; 9 Marine Biological Association, The Laboratory, Citadel Hill, Plymouth, PL1 2PB UK; 10 School of Ocean and Earth Science, University of Southampton, Southampton, UK; 11 Bioorganic Analytics, Institute for Inorganic and Analytical Chemistry, Friedrich Schiller University Jena, Lessingstr. 8, 07743 Jena, Germany

**Keywords:** Cellular microbiology, Transcriptomics, Microbial ecology

## Abstract

Sexual reproduction is a fundamental phase in the life cycle of most diatoms. Despite its role as a source of genetic variation, it is rarely reported in natural circumstances and its molecular foundations remain largely unknown. Here, we integrate independent transcriptomic datasets to prioritize genes responding to sex inducing pheromones (SIPs) in the pennate diatom *Seminavis robusta*. We observe marked gene expression changes associated with SIP treatment in both mating types, including an inhibition of S phase progression, chloroplast division, mitosis, and cell wall formation. Meanwhile, meiotic genes are upregulated in response to SIP, including a sexually induced diatom specific cyclin. Our data further suggest an important role for reactive oxygen species, energy metabolism, and cGMP signaling during the early stages of sexual reproduction. In addition, we identify several genes with a mating type specific response to SIP, and link their expression pattern with physiological specialization, such as the production of the attraction pheromone diproline in mating type − (MT−) and mate-searching behavior in mating type + (MT+). Combined, our results provide a model for early sexual reproduction in pennate diatoms and significantly expand the suite of target genes to detect sexual reproduction events in natural diatom populations.

## Introduction

Sexual reproduction is a virtually universal feature in the life cycle of eukaryotic organisms and a wealth of reproductive strategies have evolved across different phyla [1]. Likewise, sexual reproduction is an essential phase in the diplontic life cycle of most diatoms, an extraordinarily diverse group of microalgae that play an important role in primary production and biogeochemical cycling in the oceans [2, 3]. Their unique life cycle is characterized by cell size reduction during vegetative growth [4]. Cells become sexually active once their size is below a species-specific sexual size threshold (SST). Sexual reproduction restores the maximum cell size by expansion of the zygote to form large auxospores that release an initial cell [4]. Although the conservation of meiotic genes and population genetic data on sexual homologous recombination [5–7] suggest that sexual reproduction occurs in natural diatom populations, reports on sexual events remain scarce and are usually restricted to field sites with high frequency monitoring [8–11]. Successful crossing of diatoms in laboratory conditions, however, revealed a diverse range of life cycle strategies with unique features for centric, araphid pennate, and raphid pennate diatoms [4, 12].

**Fig. 1:**
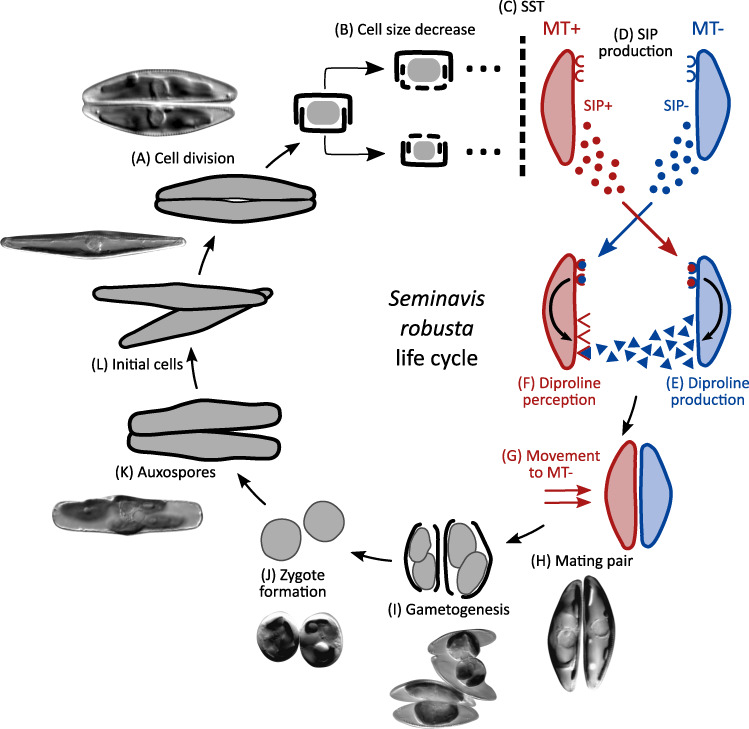
The life cycle of *Seminavis robusta*. The *S. robusta* life cycle is diplontic and consists of long periods of vegetative division alternating with short periods of sexual reproduction. **a** The average cell size decreases with every mitotic division. **b** Transverse view of a vegetative cell showing the mechanism of cell size decrease. **c** When populations pass the sexual size threshold (SST) at a cell size of about 50 µm, cells become capable of sexual reproduction. **d** Mating type + (MT+) and mating type − (MT−) start producing sex inducing pheromones called SIP+ and SIP−, respectively. SIP induces a cell cycle arrest in the compatible mating type. **e** In response to SIP+, MT− secretes an attraction pheromone: the diketopiperazine diproline, while (**f**) MT+ becomes sensitive to diproline and glides toward the diproline source. **g, h** Diproline signaling leads to mate finding and pair formation. **i, j** Each partner produces two gametes that fuse with the gametes of the compatible mating type to form zygotes. Finally, auxospores (**k**) will enlarge and release an initial cell of the original cell size (**l**). This figure was modified from Moeys et al. (2016) and Gillard et al. (2012) and some microscopic pictures were obtained from Chepurnov et al. (2002) with permission.

Characteristic for pennate diatoms, sexual reproduction is initiated by the interaction of sexually mature vegetative cells (gametangia) from compatible mating types [4]. Whereas passive physical forces are thought to steer cell–cell interaction in planktonic species [13], most benthic raphid diatoms actively move toward a partner of the compatible mating type [4]. Recently, experimental evidence has shown that certain pennate diatoms deploy sex pheromones to recognize and localize a suitable partner [14–17]. Furthermore, a multistage pheromone cascade was discovered in the raphid pennate diatom *Seminavis robusta* (Fig. 1), emphasizing the largely unexplored complexity of mate localization and recognition in diatoms [16]. Previous studies have briefly addressed the transcriptomic response to sex inducing pheromones (SIPs) in pennate diatoms [16, 17], but a detailed overview and timing of expression is currently lacking. Importantly, while the previously discovered multistage pheromone cascade suggests a mating type specific response to SIPs, it is unknown how this is reflected at the molecular level.

Here, we use RNA-sequencing (RNA-seq) to provide a detailed description of the response to SIPs in *S. robusta*. Responses in gene expression were compared between a newly generated time-series RNA-seq dataset for mating type + (MT+) and existing datasets for the compatible mating type − (MT−). We relate physiological changes resulting from a G1 phase arrest to differentially expressed genes throughout the cell cycle and show that SIP− increases motility of the attracted MT+. To tackle technical challenges in comparing gene expression between datasets, a workflow is introduced to integrate RNA-seq datasets. This approach allowed the identification of key genes exhibiting either mating type specific or shared responses to SIP. These key genes include a sexually induced cyclin and highlight the importance of reactive oxygen species (ROS), energy metabolism, and ubiquitination in the mating process.

## Material and methods

### Culture conditions

*Seminavis robusta* strains were obtained from the BCCM/DCG diatom culture collection at Ghent University (http://bccm.belspo.be/about-us/bccm-dcg, see Supplementary Table 1 for an overview of used strains) and were grown in sterile filtered natural sea water from the North Sea enriched with Guillard’s F/2 solution, except for the experiments involving MT+ motility and MT− flow cytometry where cultures were grown in artificial sea water with F/2 solution. Prior to the experiments, cultures were made axenic by adding 500 mg/L penicillin, 500 mg/L ampicillin, 100 mg/L streptomycin and 50 mg/L gentamycin to the medium for one week before the experiment. Cultures were grown at 18 °C in 12 h:12 h light:dark cycles under cool white fluorescent lamps unless stated otherwise.

### Preparation of SIP containing filtrate

MT− cultures (strain 85B) and MT+ cultures (strain D6) below the SST were cultured in 150 mL cell culture flasks for 1 week. When cultures reached the late-exponential growth phase the medium was vacuum filtered using a Stericup with pore size of 0.22 µm (Merck GmbH, Germany) in order to obtain a filtrate containing SIP− and SIP+, respectively. The potency of the filtrate was assessed using a cytokinetic arrest and a diproline attraction assay (see Supplementary Methods). The SIP filtrate was used for the RNA-seq experiment, for cell cycle analysis using flow cytometry and for assessing its effect on motility of MT+ (Supplementary Methods).

### RNA-seq experimental setup, data analysis and functional interpretation

RNA-seq data generated in this study representing the response of MT+ (strain 85A) to SIP− filtrate was complemented with existing data on the response of MT− to a chromatographic fraction containing SIP+ (time points 15 min, 1 h, 3 h) [16] and SIP+ filtrate (time point 10 h) [18]. The experimental setup for the two MT− RNA-seq datasets are described in their respective publications [16, 18]. For the novel MT+ dataset, we harvested control cultures in the dark (time point 0 h) and further harvested dark-synchronized cultures consisting of three control replicates and three SIP− treated replicates at five time points: 15 min, 1 h, 3 h, 6 h and 9 h. Details about the experimental setup are described in Supplementary Methods.

All three RNA-seq datasets were mapped to gene models from the *S. robusta* genome v1.0 (available at https://bioinformatics.psb.ugent.be/orcae/overview/Semro) [19] using Salmon v0.9.1 [20] (Supplementary Fig. 1A) and differential expression (DE) analysis was performed using negative binomial models implemented in the edgeR package [21]. Details about the model, design matrix, and contrasts of interest are described in Supplemenatry Methods. Functional annotation for all genes was derived using an ensemble of three methods: InterProScan [22], AnnoMine [23] and eggNOG-mapper [24]. Gene families were computed by clustering protein sequences with TRIBE-MCL [25]. Specific details on the construction of functional annotation are described in Supplementary Methods.

To identify biologically relevant DE genes, we used two complementary approaches, both of which are based on the same statistical model. First, we used the results from the conventional DE analysis to identify genes and biological processes involved in sexual reproduction, based on the genes’ functional annotation and the current literature. Second, we developed a novel integrative workflow that allowed us to compare the response to SIP in different RNA-seq datasets from both mating types, by testing against a fold change cut-off. We restricted the comparison to the time points that are available for both mating types (15 min, 1 h and 3 h). Genes discovered by the integrative analysis represent key genes with a shared versus mating type specific response to SIP. Deriving both analyses from the same statistical model implies that genes discovered by the more stringent fold-change analysis are also discovered in a conventional analysis. Three sets of genes were defined: genes responding to SIP in both mating types (SRBs: “SIP Responsive in Both mating types”), genes with a MT− specific response (SRMs: “SIP Responsive in mating type Minus”) and genes with a MT+ specific response (SRPs, “SIP Responsive in mating type Plus”). Details about the integrative workflow can be found in Supplementary Methods.

### Data availability and reproducibility

The raw data from the new RNA-seq experiments are available at the European Nucleotide Archive (ENA) at EMBL-EBI under accession number PRJEB35793 (https://www.ebi.ac.uk/ena/data/view/PRJEB35793). Additional genomic information concerning genes mentioned here can be found on the diatom PLAZA platform for comparative genomics (https://bioinformatics.psb.ugent.be/plaza/versions/plaza_diatoms_01/). All scripts required to reproduce the analyses and figures reported in this paper as well as Salmon estimated count matrices and results of statistical tests are available on our GitHub repository at https://github.com/statOmics/SeminavisComparative.

## Results and discussion

**Fig. 2:**
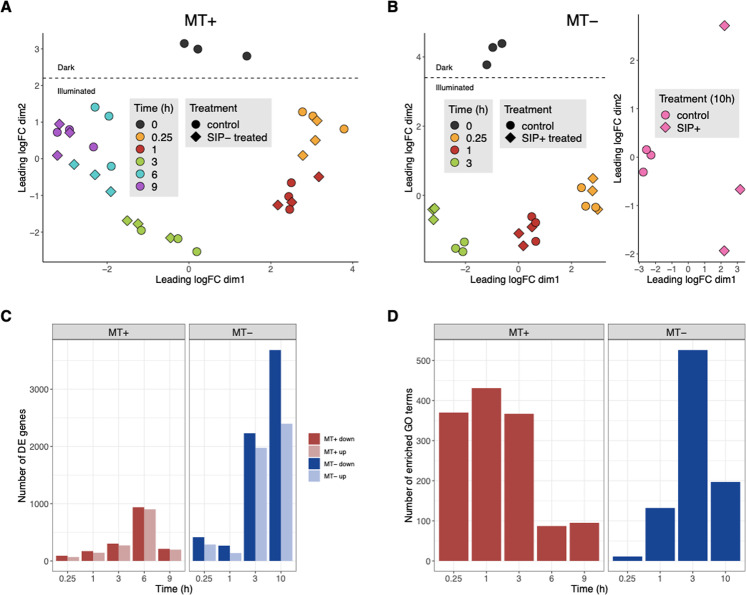
Transcriptional responses induced by SIP treatment of *Seminavis robusta*. **a** Multidimensional scaling (MDS) plot for MT+ expression data (0 h–9 h), and (**b**) MDS plots of two MT− expression datasets (0 h–3 h and 10 h, respectively). Distances between samples in the MDS plot approximate the log2 fold changes of the top 500 genes. **c** Number of significant DE genes between control and SIP treated cultures for each time point in both mating types. Each dataset was analyzed on a 5% overall FDR (OFDR) level, i.e., the fraction of false positive genes over all rejected genes. The color represents mating type (MT+: red, MT−: blue) and the shade denotes direction of change (dark: downregulated after SIP treatment, light: upregulated after SIP treatment). **d** Number of significantly enriched GO terms on a 5% FDR level for each time point. The color represents mating type (MT+: red, MT−: blue). A high number of GO terms are discovered in the early time points for MT+, in comparison to the number of DE genes.

In this study, we generated a new time-series dataset to investigate the response of dark-synchronized MT+ cultures to SIP− filtrate (6 time points, 0–9 h, Fig. 2a) and to compare expression patterns with two existing datasets on the response of MT− to SIP+ [16, 18] (4 time points, 0–10 h, Fig. 2b). Here, we first report the results of a conventional DE analysis for each dataset separately. Next, we use these results to discover genes or biological processes related to sexual reproduction, either based on their predicted functional annotation or the current literature. Finally, we integrate the results of both the novel and publicly available datasets in an integrative analysis that aims at identifying key genes involved in either a single or both mating types.

### General transcriptional response and identification of key SIP responsive genes

Multidimensional scaling plots of the RNA-seq data (Fig. 2a, b) showed that in both mating types the dark-to-light transition and time since illumination were the major drivers of gene expression change throughout the experiments. However, as time progresses, the effect of SIP becomes more pronounced. This is supported by DE analysis showing that the number of significant genes increased markedly at later time points (Fig. 2c). Overall, most genes are significantly DE in only a single time point on a 5% overall FDR (OFDR) level (Supplementary Fig. 1B). Combined, on a 5% OFDR level, 4037 genes are DE in response to SIP treatment for MT+, while 5486 genes are found to be DE in MT− in the first 3 h and 6079 genes after 10 h. The stronger response during the first 3 time points in MT− versus MT+ may be the result of the use of a chromatographic fraction of SIP+ for MT− while MT+ cultures were treated with a SIP− containing filtrate. Gene sets for many biological processes were significantly enriched in the conventional lists of DE genes: a total of 1081 and 740 enriched biological process terms were discovered for the response to sex pheromones in MT+ and MT−, respectively (Fig. 2d, Supplemenatry Fig. 2).

**Fig. 3:**
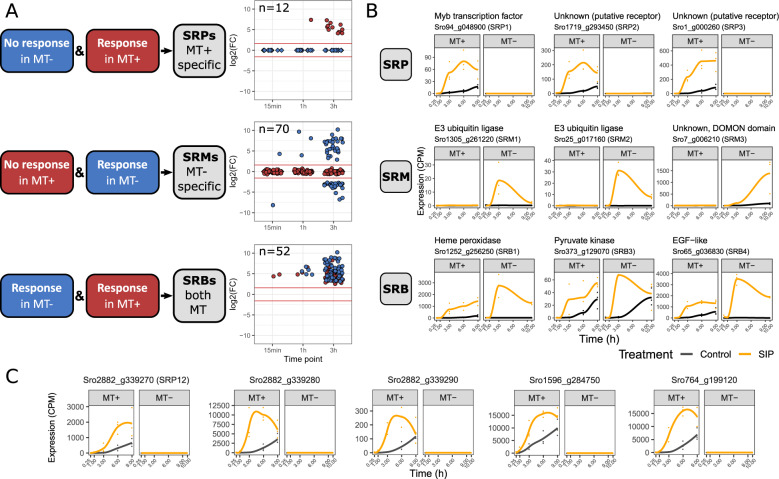
Visualization and main results of the integrative workflow. **a** Schematic representation of the integrative workflow indicating how SIP responsive genes with a shared response (SRBs) or mating type specific response (SRPs, SRMs) were discovered. Non-responsive genes consist of genes that are equivalently expressed after SIP treatment versus control, or that are very lowly/not expressed (filtered). A log fold change (LFC) cutoff of ±log(3) was used to define responsive (differentially expressed) genes and equivalent genes. At the right side of the panel, log2 fold changes of SRMs, SRPs and SRBs in both mating types are plotted. Each gene is plotted for the time point at which they are differentially expressed. Genes which were not expressed (“filtered”) in the non-responsive mating type are plotted as diamonds. The red horizontal lines represent the fold change cutoff used to determine equivalence and differential expression. The number of discovered genes is indicated in the top left corner of each plot. **b** Expression of a selection of SIP responsive genes (SRMs, SRPs, SRBs). For each gene, counts per million (CPM) are plotted as a function of time for both mating types. The data points correspond to gene expression of the replicates in each time point and the solid line represents the mean. Data points and lines are colored according to condition, i.e., black for control condition and orange for SIP treatment. **c **Expression of the five genes belonging to the gene family of SRP12 (*Sro2882_g339270*). Data are presented in the plots in the same manner as in (**b**).

We discovered key genes by developing a statistical integrative analysis workflow that is capable of testing for equivalent, i.e., non-DE, expression between conditions. Coupling equivalence testing in one mating type with DE calls in the other allowed for the discovery of key genes exhibiting mating type specific responses to SIP, while DE calls in both datasets found key genes responsive in both mating types. This workflow revealed 52 key genes responding to SIP in both mating types (SRBs), 12 genes uniquely responding in MT+ (SRPs) and 70 genes uniquely responding in MT− (SRMs) (Fig. 3a, Supplementary Figs. 3, 4, 5). Similar to the conventional DE analysis, the response of MT− was more pronounced compared to MT+, likely due to technical differences such as different protocols for pheromone administration. Remarkably, while in MT− we discovered a comparable number of down- and upregulated SRMs, we only found upregulated SRPs and SRBs, possibly indicating that sexual processes induced by SIPs are mainly driven by the induction of key genes rather than the downregulation of inhibitory genes (Fig. 3a).

**Fig. 4:**
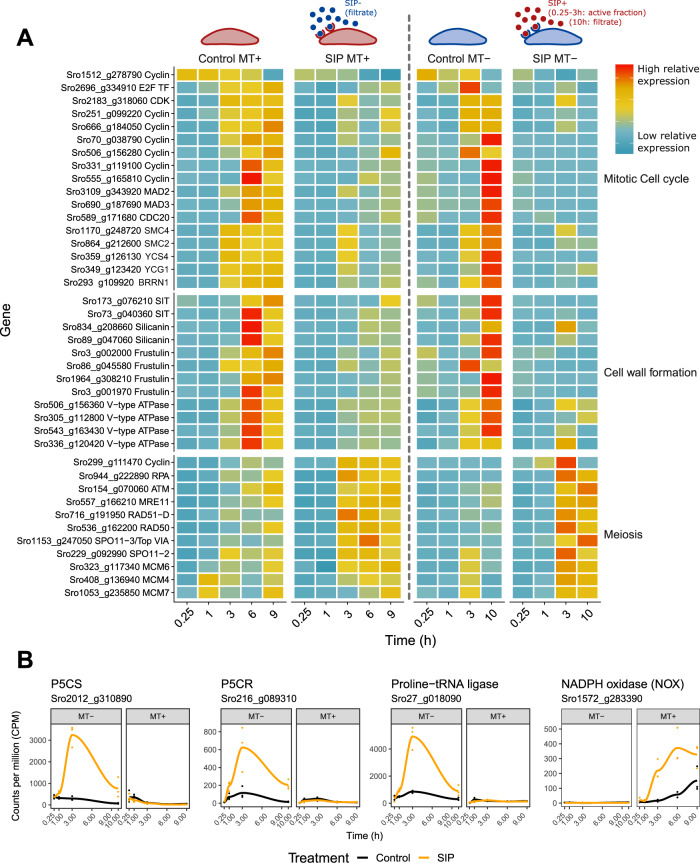
Gene expression of *Seminavis robusta* genes involved in mating-related processes. **a** Heatmap of genes related to mitotic and meiotic cell cycle progression, which are differentially expressed (DE) in both mating types in the conventional DE analysis. Each gene is plotted for control and SIP treated conditions in both *S. robusta* mating types. Genes are specified as row names and are scaled relative to the mean expression, amounting to counts per million (CPM) standardized to zero mean and unit variance for each gene in each mating type separately. Blue indicates low expression, while red indicates high expression. **b** Expression of genes related to diproline synthesis and reactive oxygen species (ROS) production, which are significantly DE in only one mating type in the conventional DE analysis. CPM are plotted as a function of time for both mating types. The data points correspond to gene expression of the replicates in each time point while the solid line represents the mean. Data points and lines are colored according to condition, i.e., black for control condition and orange for SIP treatment. P5CS = Δ1-pyrroline-5-carboxylate synthetase; P5CR = Δ1-pyrroline-5-carboxylate reductase.

In what follows we will discuss in more detail the genes and pathways that are responding to SIP in both mating types or uniquely in one mating type and link these changes to physiological events in the mating process. In each section, we first discuss key genes highlighted by the integrative analysis (Fig. 3), after which we discuss results from the conventional DE analysis, focussing on selected biological processes (Fig. 4).

### Responses to SIP conserved in both mating types

#### Integrative analysis reveals key genes responsive in both mating types

A large fraction (22/52) of SRBs, i.e., key genes with a strong response to SIP in both mating types, lack any functional annotation and homology to sequenced genomes of other diatoms (Supplementary Table 2), suggesting that the molecular mechanisms underlying early mating are highly species-specific. The remaining 30 SRBs can be linked to energy metabolism, ROS signaling and meiosis, amongst others. Pyruvate kinase (*Sro373_g129070*) and isocitrate dehydrogenase (*Sro492_g153950*), respectively involved in glycolysis and the citric acid cycle, are strongly upregulated in both mating types (Fig. 3b), suggesting an increased energy demand. Interestingly, pyruvate kinase is also upregulated during gametogenesis in the brown alga *Saccharina latissima* [26] and the parasite *Plasmodium berghei* [27]. In addition, two enzymes from the pentose phosphate pathway (PPP) are among the SRBs: transketolase (*Sro524_g159900*) and transaldolase (*Sro196_g083630*) (Supplementary Fig. 3). The PPP generates NADPH, a reductive compound needed in various metabolic reactions and involved in detoxification of ROS by regenerating glutathione [28, 29]. Furthermore, one SRB encoding a heme peroxidase (*Sro1252_g256250*) exhibited strong upregulation upon SIP treatment (Fig. 3b). Upregulation of heme peroxidases was also reported during sexual reproduction in other eukaryotes, e.g., mosquitoes (*Anopheles gambiae*) [30] and fungi [31, 32]. Heme peroxidases promote substrate oxidation in various metabolic pathways and are essential for the detoxification of ROS [33], suggesting that ROS signaling plays a role in the response to SIP, as seen in the green algae *Volvox carteri*, where high ROS levels trigger sex [34]. Finally, a highly expressed SRB encodes a transmembrane protein containing an Epidermal Growth Factor-like (EGF-like) domain (*Sro65_g036830*, Fig. 3b), with potential orthologs encoded in pennate and centric diatoms including *P. tricornutum* and *T. pseudonana* (BLASTp, E < 1e-10). EGF-like domains are generally extracellular protein modules that play a role in receptor/ligand interactions, intracellular signaling, and adhesion [35]. Membrane bound proteins containing EGF-like domains are required for gamete fusion in green algae [36] and oocyte binding by animal sperm cells [37–39]. Furthermore, multiple EGF-like repeats were discovered in sexually induced genes Sig1–3 of the centric diatom *Thalassiosira weissflogii* [40]. Sig1 orthologs were later shown to be located on the mastigonemes of stramenopile flagella [41], suggesting their upregulation is related to the differentiation of flagellated male gametes in centric diatoms. We could not detect orthologs of Sig1–3 in the genome sequence of *S. robusta* (BLASTp, E < 0.001), in line with the lack of flagellated stages in pennate diatoms. Nevertheless, the presence of EGF-like domains in sexually induced genes in pennate and centric diatoms suggests that genes containing EGF-like domains play a role in diatom cell–cell communication or gamete fusion.

#### Conventional DE analysis uncovers mating-related processes differentially regulated in both mating types

**Fig. 5:**
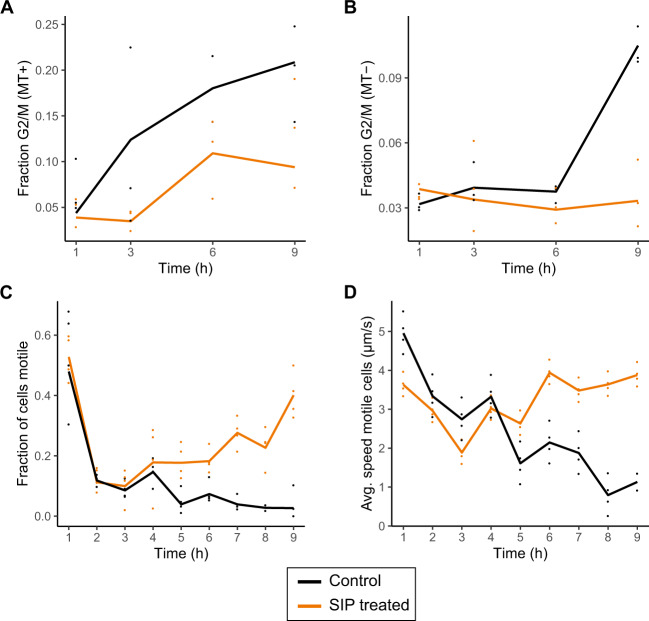
Physiological responses to SIP. **a, b** The fraction of cells in the G2 or M phase during the RNA-seq experiment for MT+ (**a**), and for MT− (**b**) as determined by flow cytometry (*n* = 3). For both MT+ and MT−, SIP induces a G1 phase cell cycle arrest, apparent by a significant lower fraction of cells in G2 and M phase after 3 h and 9 h (MT+) and 9 h (MT−). **c, d** Motility of MT+ cells over time after treatment with a 1/10 dilution of SIP filtrate (*n* = 4). Both the proportion of motile cells in the culture was determined (**c**) as well as the gliding speed of cells that are motile (**d**). Note that an initial high proportion of motile cells are observed after 1 h in all treatments, likely the result of a phototactic response following illumination after a prolonged period of darkness. For all panels, points show individual data points while solid lines show the average per time point. Untreated (control) cultures are indicated in black, while SIP treated cultures are represented in orange.

We used flow cytometry to confirm a sex pheromone induced G1 phase arrest, which was proposed for *S. robusta* and *Pseudo-nitzschia multistriata* [16, 17]. Treatment with SIP significantly decreased the proportion of G2/M phase cells in MT+ after 3 h (control 12.4% versus SIP 3.5%, *p* = 0.0023) and 9 h (control 20.9% versus SIP 9.4%, *p* = 0.0123) and after 9 h in MT− (control 10.5% versus SIP 3.3%, *p* < 0.0001) (Fig. 5a, b). A temporary arrest of the cell cycle appears to be a prerequisite for a switch to meiosis in many diatoms [16, 17, 42, 43], although in the centric diatom *Skeletonema marinoi* no growth arrest was observed during sexual reproduction [44]. The cell cycle arrest is reflected in the transcriptomic data as a sequential downregulation of cell cycle genes in the conventional DE results (Fig. 4a), causing an enrichment in cell cycle related GO terms (Supplementary Fig. 2). In eukaryotes, S phase progression is controlled by E2F transcription factors forming a heterodimer with Dimerization Partner (DP) transcription factors [45]. In accordance with plants and animals [46, 47], we observe an increase in expression of E2F transcription factors in control conditions as cells go through the cell cycle. SIP treatment significantly repressed expression of two E2Fs (*Sro2696_g334910* and *Sro1798_g298290*) in both mating types and MT−, respectively (Supplementary Fig. 6A). The transcriptional repression of E2Fs is likely caused by the inability of cells to enter S phase as a result of the G1 phase arrest, although the activity of E2Fs is generally also regulated by other mechanisms such as Retinoblastoma-related (Rb) protein binding and phosphorylation which we did not assess [46, 47]. Furthermore, two DP genes were DE in MT− in response to SIP+; one (*Sro905_g218540*) was repressed, while the other (*Sro905_g218570*) was induced, suggesting they play a contrasting role in the cell cycle arrest (Supplementary Fig. 6A).

During the S and G2 phase of the mitotic cell cycle, fission of both chloroplasts of *S. robusta* results in four daughter chloroplasts by the start of the M phase [48]. FtsZ and dynamin-related protein 5B (DRP5B) are key factors in the formation of a multi-ring structure that constricts the chloroplast during fission [49, 50]. In control conditions, we observe a expression peak of two FtsZ homologs (*Sro1409_g270150* and *Sro931_g221490*) and DRP5B (*Sro814_g206290*), potentially coinciding with the timing of chloroplast division in *S. robusta* [48] (Supplementary Fig. 6B). After exposure to SIP, expression of these genes was significantly repressed in both mating types (Supplementary Fig. 6B), indicating that the cell cycle arrest results in an inhibition of chloroplast fission. Indeed, chloroplast division ceases during sexual reproduction of *S. robusta* so that each gamete will contain one chloroplast and the auxospore inherits two chloroplasts [51]. Noteworthy, while DRP5B is restricted to the chloroplast [52], stramenopiles can encode a mitochondrium-targeted FtsZ that is involved in mitochondrial division [53]. Thus, it is possible that the downregulation of FtsZ genes is linked with mitochondrial instead of chloroplastic division.

In the transcriptomic data, seven cyclins were downregulated in both mating types after treatment with SIP (Fig. 4a). Six out of seven appear in a cluster of genes, which peak late in the time series in control conditions (6 h, 9 h and 10 h; Supplementary Fig. 7), suggesting that they are mitotic cyclins involved in G2/M transition [54]. To determine which cyclin family they represent, a maximum likelihood phylogenetic tree of *S. robusta* cyclins was constructed (Supplementary Fig. 8). The repressed cyclins consist of three diatom-specific cyclins (dsCyc), three A/B type cyclins and one cyclin D (Supplementary Table 3).

Several genes involved in mitosis were downregulated in both mating types, including MAD2 (*Sro3109_g343920*), MAD3 (*Sro690_g187690*) and CDC20 (*Sro589_g171680*), which form the Mitotic Checkpoint Complex. Furthermore, we observe downregulation of five genes coding for subunits of the condensin complex, which play a central role in chromosome organization during mitosis and meiosis [55] (Fig. 4a).

Finally, at the end of each mitotic cell cycle, diatom cells produce a new valve in a silica deposition vesicle (SDV) prior to cytokinesis. As it was previously shown [16], treating cultures with SIP of the compatible mating type reduces the fraction of cytokinetic cells after 14 h (Supplementary Fig. 9A, *p* < 0.0001 for both MT+ and MT−). Accordingly, we observed downregulation of genes known to be important for silica cell wall formation after treatment with SIP (Fig. 4a), including two out of five *S. robusta* silicic acid transporter homologs, involved in the uptake of silicic acid from the environment [56]. In addition, we find two (out of a total of three) silicanin1-like genes as well as four (out of thirteen) frustulins, respectively proteins embedded in the SDV membrane and proteins which are found in the organic casing surrounding the cell wall [57, 58]. Moreover, four genes making up various subunits of V-type ATPase complexes were significantly downregulated in both species upon treatment with SIP. These proton pumps play a role in biomineralization of silica by acidifying the SDV [59].

While mitotic cell cycle genes were generally downregulated by SIP treatment, we observed an increase in meiotic gene expression, accompanied by an enrichment in the “meiotic chromosome condensation” GO term in both mating types (Supplementary Fig. 2). Surprisingly, one cyclin which is an SRB (*Sro299_g111470*, Fig. 4a) is upregulated rather than downregulated in response to the pheromone. Its expression pattern suggests it plays a role either in SIP induced physiological responses such as mate finding or in preparing the cell for meiotic cell cycle progression. The gene has presumably evolved independently from the sexually induced cyclins of other major eukaryotic clades, as phylogenetic analysis places the gene among diatom specific cyclins (dsCyc) (Supplementary Fig. 8). The expansion of the cyclin family in diatoms compared to other members of the SAR clade [60] might have been instrumental to allow the diversification of this sexually induced cyclin [61–64].

To investigate the dynamics of other meiotic genes throughout the experiment, we explored the expression of a set of 42 known meiotic and bifunctional mitotic/meiotic diatom genes [5], some of which were induced in response to sex pheromones in pennates [16, 17] and during sexual reproduction in the centric diatom *S. marinoi* [44]. Here, we found ten meiotic markers significantly responding to SIP in at least one time point in both mating types (Fig. 4a). These include genes encoding DNA replication licensing factors MCM4, MCM6, MCM7, which are involved in the initiation of replication during the mitotic and meiotic S phase [5]. Two homologues of SPO11 (SPO11–2 and SPO11–3) are upregulated in both mating types. SPO11 plays a role in the formation of double strand DNA breaks during homologous recombination. In diatoms and plants, the SPO11–2 homologue was shown to be meiotic while SPO11–3 is involved in vegetative growth [5, 17, 65]. However, the observed upregulation of SPO11–3 in response to SIP in *S. robusta* and during sexual reproduction in the centric *S. marinoi* [44] suggest that in addition to SPO11–2, SPO11–3 might also be involved in meiotic homologous recombination in diatoms. Three genes involved in DNA repair after the induction of double stranded breaks by SPO11 were upregulated in both mating types: MRE11, RAD50, and RAD51D [5]. Finally, we observed the upregulation of two genes not yet described in the meiotic toolkit of diatoms: Ataxia Telangiectasia Mutated (ATM, *Sro154_g070060*) which codes for a protein that controls double-strand break formation by SPO11 during meiosis [66] and Replication Protein A (RPA, *Sro944_g222890*), involved in the binding of ssDNA during replication and homologous recombination [67] (Fig. 4a). Interestingly, although meiotic genes are upregulated in response to SIP, the G1 phase arrest inhibits progression through the meiotic cell cycle and we did not observe gametogenesis in MT+ cells attracted to diproline loaded beads. Accordingly, chloroplast rearrangements and indications of the meiotic prophase were only observed microscopically after compatible cells form a mating pair [51]. We therefore hypothesize that a separate, local signal during cell pairing is required to break the G1 phase arrest and induce gametogenesis.

In response to SIP, 17 genes with a guanylate cyclase domain (GC) were significantly DE in both mating types, 8 of which form a bifunctional guanylate cyclase/phosphodiesterase (GC/PDE) fusion enzyme. Interestingly, the GC and PDE domains in these genes have contrasting functions, respectively synthesizing and breaking down the secondary metabolite cGMP. Although the genes with only a GC domain do not show a general direction of regulation, all GC/PDE genes were upregulated (Supplementary Fig. 10), including *Sro991_g228730*, which was previously described to be responding to SIP in *S. robusta* [16]. All significant GC/PDEs show the topology described by Moeys et al. (2016) [16] a small N-terminal intracellular domain, an extracellular stretch and a long C-terminal intracellular part containing the GC and PDE domains. Thus, cGMP signaling appears to be a conserved response to sex pheromones in pennate diatoms, as guanylate cyclases are also upregulated in *P. multistriata* [17].

Finally, orthologs of three uncharacterized genes induced during sexual reproduction in the centric diatom *S. marinoi* and the pennate diatom *P. multistriata* [44], were upregulated in response to SIP (Supplementary Fig. 6C). *Sro587_g171310* (orthologous to *STRINITY_DN12692_c0_g1_i1* and *Pmu0010180*) was significantly upregulated in both mating types. Protein domain analysis revealed the presence of a Homologous-pairing protein 2 (Hop2) domain in its *S. robusta* (IPR010776)*, P. multistriata* (IPR010776) and *S. marinoi* (IPR040461) sequence. Although Hop2’s role in homologous recombination would be in accordance with the observed expression, more work is needed to confirm this gene as a Hop2 homologue, as Hop2 is presumed to be absent in diatoms [5, 44]. Another conserved sexual gene (*Sro131_g062230*, orthologous to *Pc15065_g1_i1* and *Pmu0009930*), is upregulated in response to SIP only after 10 h of SIP treatment in MT−. Finally, *Sro637_g179400* (ortholog of *MTRINITY_DN9343_c0_g1_i2* and *Pmu0061540*) was upregulated after 3 h and 10 h in MT−. Interestingly, domain predictions show that the protein consists of an unknown N-terminal domain, followed by one transmembrane helix and a C-terminal beta-propeller domain in all three species (IPR013519, IPR011043 and IPR015943 in *S. robusta, P. multistriata* and *S. marinoi* respectively). This topology suggests a function as a receptor or in adhesion [68]. Since these three genes are upregulated in two pennate and one centric diatom species, they are interesting candidate marker genes for sexual reproduction in diatoms. Notably, we discovered five additional conserved sexual genes in the *S. robusta* genome which are not differentially expressed to SIP. If their function is conserved, we expect these genes to be upregulated during zygote or auxospore formation rather than during pheromone signaling, since the physiology of mate finding strongly differs between species.

### Responses to SIP specific for MT+

We identified 12 genes displaying a MT+ specific response to SIP (SRPs, Fig. 3a, Supplementary Table 2), among them a Myb-like transcription factor (*Sro94_g048900*), which is likely regulating downstream events associated with the perception of SIP− by MT+. One such downstream response unique for MT+ is responsiveness to the attraction pheromone diproline [16], which we confirmed using a bead attraction assay (Supplementary Fig. 9B). This might be caused by SIP− induced expression of a diproline receptor in MT+. While biosynthetic pathways of 2,5-diketopiperazine cyclodipeptides such as diproline have been elucidated, cyclodipeptide receptors are not well characterized [69]. Among the SRPs, we have identified two functionally unidentified genes which satisfy the requirements for a potential diproline receptor: *Sro1719_g293450* and *Sro1_g000260*. Both encode transmembrane proteins and show high expression uniquely in response to SIP− in MT+ (Fig. 3b). Interestingly, the former gene is predicted to contain 7–8 transmembrane helices, reminiscent of G-protein coupled receptors (GPCR) which often associate with cyclodipeptides in humans [69] and, moreover, a GPCR was upregulated in response to sex pheromones in *P. multistriata* [17]. Sexual *S. robusta* MT− cells were shown to predominantly secrete the cyclo(L-Pro-L-Pro) enantiomer [14]. However, while pheromone receptors are usually stereosensitive, synthetic cyclo(D-Pro-D-Pro) is also biologically active, suggesting a highly unusual stereo-insensitive receptor or racemization of the pheromone [14, 70]. Further, the SRPs include 6 genes lacking functional annotation, among which is SRP12 (*Sro2882_g339270*) that belongs to a gene family containing five *S. robusta* genes of which three are located adjacently on the same genomic contig (*Sro2882_g339270, Sro2882_g339280* and *Sro2882_g339290)*. Four out of five show a MT+ specific upregulation to SIP− with very high expression ranging up to 1.5% of the total transcriptome library (Fig. 3c). Furthermore, all five genes exhibit a high baseline MT+ expression and negligible expression in control MT− conditions (Fig. 3c), suggesting they play a role in mating type differentiation, comparable to MRP genes in the pennate *P. multistriata* [71].

When exploring ROS signaling related genes, the conventional DE analysis uncovered an NADPH oxidase (NOX, Sro1572_*g283390*) with a pronounced MT+ specific response to SIP (Fig. 4b), catalyzing extracellular production of superoxide anions by moving an electron from NADPH through the plasma membrane to molecular oxygen. The observed NOX contains six transmembrane domains [72] and an EF-hand domain suggesting a potential link with calcium signaling. As superoxide is cell impermeable, extracellular superoxide is most likely dismutated to H_2_O_2_, which can enter neighboring cells for ROS signaling, e.g., through aquaporin channels [73, 74]. NOX activity during sexual reproduction is a common theme in eukaryotes: it is required for gametogenesis and fertilization in plants [75] and is required for the formation of sexual fruiting bodies in the fungus *Aspergillus nidulans* [32]. Furthermore, gametophytes of the kelp *S. latissima* show female specific expression of NOX, suggesting mating type specific expression of NOX during sexual reproduction may be conserved among stramenopiles [26]. Notably, enzymes from the NADPH producing PPP were upregulated in both mating types, potentially supplying NADPH to support NOX-mediated superoxide production.

To assess whether SIP− affects the motility of MT+, the displacement of control and SIP− filtrate treated cells was tracked for 30 sec over the course of 9 h (Supplementary Fig. 11). We observed mate-searching behavior starting 6 h–7 h after treatment with SIP−. Not only did the fraction of motile cells (i.e., cells showing any displacement) in the culture increase (Omnibus test *p* < 0.001, Fig. 5c, Supplementary Fig. 12A), the speed of the subset of motile cells also increased: after 9 h, the average speed of motile SIP− treated cells was 3.8 µm/s compared to 1.1 µm/s for untreated cells (Omnibus test *p* < 0.0001, Fig. 5d, Supplementary Fig. 12B). Thus, exposure to SIP does not only prime MT+ cells to become responsive to the attraction pheromone diproline, the increased motility of gametangia further increases the probability to encounter an immotile, diproline producing MT− cell. Furthermore, the pre-activation of cellular motility machinery by SIP− might explain the almost immediate attraction to a new diproline source [76]. The timing of the mating behavior in *S. robusta* appears to be remarkably synchronized between mating types: significant increases in motility occurred simultaneously with the first noticeable amount of diproline produced by MT− [14] and the onset of responsiveness of MT+ to diproline (Supplementary Fig. 9B). As cell motility in raphid diatoms is achieved through the secretion of adhesive molecules that attach the cell to the substratum and provide traction for their gliding movement [77], we performed BLAST searches to identify adhesive proteins containing a GDPH-domain, named after a conserved Gly-Asp-Pro-His amino acid motif [78]. Among 87 GDPH-containing proteins identified, four are upregulated in MT+ in response to SIP− treatment in the conventional DE analysis (Supplementary Fig. 6D). However, three out of four GDPH-domain-containing genes are also significantly upregulated in immotile SIP+ treated MT− cells and one is upregulated uniquely in MT−. Therefore, as previously suggested, the GDPH-domain containing proteins may play additional roles related to extracellular adhesion in addition to cell motility, such as mucilage pad, stalk and chain formation as well as cell–cell adhesion during mating [78].

### Responses to SIP specific for MT−

A relatively high number (70) of SRMs, i.e., genes with a MT− specific response to SIP, were discovered (Fig. 3a) of which 16 lack any functional annotation (Supplementary Table 2). Molecular functions of SRMs are diverse, including receptors, membrane channels, guanylate cyclases and other signaling enzymes. Among the SRMs are two E3 ubiquitin ligase genes (*Sro1305_g261220* and *Sro25_g017160*) representing the Ring-Between-Ring (RBR) and U-box family, respectively. Since ubiquitin ligases are important players in signaling by targeting downstream proteins for ubiquitination [79], these genes are potential key regulators of MT− specific responses such as the production of diproline. Ubiquitin ligases also play a role in meiosis across eukaryotes by targeting proteins for proteasomal degradation [80]. However, a function in meiosis for the identified ubiquitin ligases is hard to reconcile with their mating type specific response, as meiosis occurs in both partners. SRMs further include a gene with a DOMON domain, which is a presumed heme or sugar sensor domain (*Sro7_g006210*) [81] (Fig. 3b). Interestingly, two SRM genes belong to an unknown and *S. robusta* specific gene family containing a zinc finger domain (Supplementary Fig. 6E).

Our conventional DE analysis also shows that glutamate-to-proline conversion enzymes Δ1-pyrroline-5-carboxylate synthetase (*Sro2012_g310890*, P5CS), and Δ1-pyrroline-5-carboxylate reductase (*Sro216_g089310*, P5CR) were significantly upregulated after SIP treatment in MT− after 3 h and 10 h, while in MT+ both genes were not significantly responding (Fig. 4b). The unique increase of proline biosynthesis in MT− is further supported by the enrichment of the “proline biosynthetic process” GO term in the 3 h time point only in MT− (Supplementary Fig. 2). Their MT− specific response supports the hypothesis that upregulation of P5CS and P5CR increases the cellular proline pool as a precursor for diproline biosynthesis [16]. Furthermore, a proline-tRNA ligase (*Sro27_g018090*) exhibits a strong significant upregulation uniquely in MT−, with expression levels exceeding 4000 CPM (Fig. 4b). This enzyme attaches proline to transfer RNA (tRNA), which serves as a substrate for ribosomal protein synthesis, explaining its consistent expression in control conditions in this dataset (Fig. 4b). The cyclodipeptide ring of diketopiperazines such as diproline is typically synthesized by either nonribosomal peptide synthetases or cyclodipeptide synthases (CDPS) [82]. Interestingly, CDPS require aminoacyl-tRNA as a substrate for the reaction instead of a free amino acid [83]. Thus, the observed MT− specific upregulation of a proline-tRNA ligase likely caters to the increased need for proline-tRNA^pro^ driving CDPS dependent diproline biosynthesis. BLAST searches in the *S. robusta* genome revealed several candidate CDPS genes, with rather low levels of conservation (Supplementary Table 4), of which none show significant regulation at any time points in any mating type. Thus, either an unidentified, transcriptionally controlled CDPS exists or one of the identified CDPS is non-transcriptionally regulated to be active only in SIP+ treated MT− cells with a cell size below the SST.

## Conclusion

**Fig. 6:**
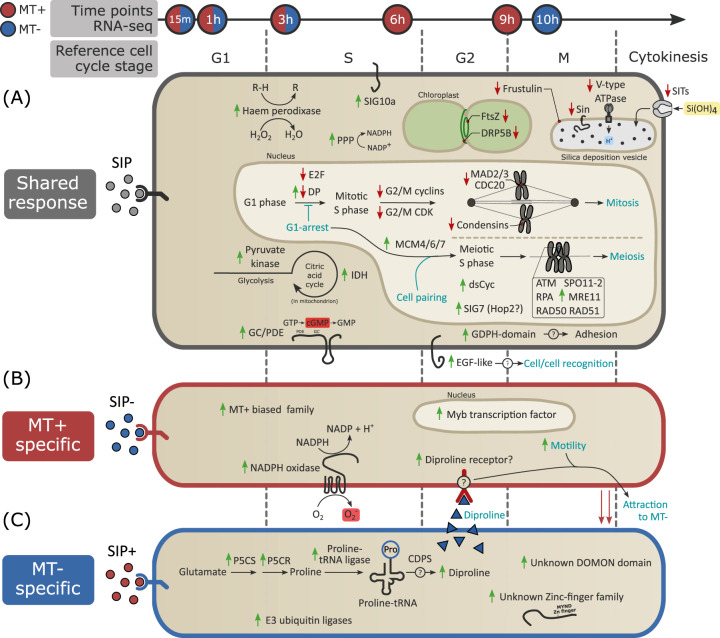
Overview of molecular and physiological processes in response to SIP in both mating types of *Seminavis robusta*. The arrow on top displays the harvest time of RNA-seq samples. Blue filled time points represent samples taken from MT−, red filled time points are samples from MT+ and red/blue filling indicates sampling for both mating types. The approximate timing of different cell cycle phases is shown below the arrow. **a** Cellular processes taking place in both mating types in response to SIP, (**b, c**) cellular responses to SIP unique for MT+ and MT−, respectively. Genes that are significantly up- or downregulated in response to SIP are depicted with a green or red arrow, respectively. Physiological events are indicated in cyan. PPP = pentose phosphate pathway, IDH = isocitrate dehydrogenase, P5CS = Δ1-pyrroline-5-carboxylate synthetase, P5CR = Δ1-pyrroline-5-carboxylate reductase and CDPS = cyclodipeptide synthase. “SIG” follows the nomenclature for conserved sexual genes used in Ferrante et al. (2019) [44].

Our study shows that, in pennate diatoms, the perception of extracellular chemical cues triggers behavioral changes and alters gene expression of gametangial cells, preparing them for cell pairing and gamete formation (Fig. 6). The response of a number of genes to SIPs was shared by both mating types, including bifunctional GC/PDE genes, an EGF-like transmembrane gene similar to sperm-egg recognition factors in animals [37–39] and several potential adhesive genes unique to diatoms [78]. Furthermore, we confirmed the induction of a G1 arrest in both mating types, reflected in a downregulation of essential genes involved in S phase progression, organelle division, mitosis and cell wall formation. To some extent, parallels can be drawn with yeasts, where compatible sex pheromones also induce a G1 phase arrest, resulting in the downregulation of key cell cycle genes [84–86]. In addition, we observed that in the diploid *S. robusta* gametangial cells of both mating types, the expression of meiotic genes was triggered by SIP, including the first sexually induced cyclin characterized in diatoms. Sex pheromone exposure in *S. robusta* thus activates both mate-finding and meiotic transcriptional programs, a feature also observed in the yeasts *Schizosaccharomyces pombe* and *Candida lusitaniae*, while mating and meiotic programs are strictly separated in the model yeast *Saccharomyces cerevisiae* [87].

Treatment with SIP also elicited mating type specific behavior and gene expression. The MT− specific response of proline biosynthesis and proline-tRNA ligase genes suggests that the synthesis of the attraction pheromone diproline is cyclodipeptide synthase (CDPS) dependent, while two SRPs encode potential diproline receptors. To our knowledge, evidence for such a complex multi-step pheromone signaling system is missing for other micro-algae. Sex pheromones are known to also induce an asymmetric expression of pheromone and receptor genes in the charophycean green alga *Closterium*, but the presence of an additional attraction pheromone remains to be confirmed [88–90]. The MT+ specific response to SIP shows some similarities with land plants, where superoxide-producing NOX also plays an important role in sexual reproduction [91, 92]. Similar to other stramenopiles, where a small set of strongly sex-biased genes underlies mating type differentiation [71, 93], we identified six genes expressed uniquely in MT+ in control conditions that are upregulated following exposure to SIP, including five members of an uncharacterized gene family and a Myb transcription factor. In plants too, male/female determining Myb transcription factors with gender specific expression are found [94–96], while they are also implicated in the sexual phase of fungi and ciliates [17, 95, 97, 98]. Finally, we report several unknown SIP responsive genes, which may include novel cell–cell communication and gamete fusion genes. Among them, genes conserved among pennate diatoms should be prime targets for further research. These potential new marker genes provide a much-needed tool for in situ monitoring of the phenology of sexual reproduction in the natural diatom population.

## Supplementary information

Supplementary Methods

Supplementary Figures

Supplementary Figure 3

Supplementary Figure 4

Supplementary Figure 5

Supplementary Table 1

Supplementary Table 2

Supplementary Table 3

Supplementary Table 4
