# Supplementary material for: Mating type specific transcriptomic response to sex inducing pheromone in the pennate diatom Seminavis robusta
Source: ISME J. 2020 Oct 7;15(2):562–76. doi: 10.1038/s41396-020-00797-7 (PMC8027222; doi:10.1038/s41396-020-00797-7)
Supplement: Supplementary file 1 — Supplementary Methods [file 41396_2020_797_MOESM1_ESM.docx]

Supplementary methods

# Testing the potency of SIP- and SIP+ filtrate

The number of mitotically dividing cells in SIP treated versus control samples was counted to assess the potency of the filtrate. Late-exponential MT+ (strain 85A) and MT- (strain PONTON34) cultures were synchronized by introducing a 36h dark period. Afterwards, respectively SIP- or SIP+ filtrate was added in three different concentrations (½, 1/200 and 1/20 000 for MT+ and ⅓, 1/10 and 1/100 for MT-). Note that a different but largely overlapping dilution series was used for each mating type. Control cultures received medium without SIP. Microscopic pictures were taken after 14h. We counted the proportion of cytokinetic cells using the Cell Counter plugin in FIJI (ImageJ). Statistical inference was carried out by modeling the expected fraction of cytokinetic cells using a quasibinomial generalized linear model (GLM) with logit link as a function of dilution. We first assessed the omnibus test, testing the null hypothesis that the expected fraction of cytokinetic cells is equal between each dilution and the control. Upon rejection of the omnibus test, a post-hoc analysis was carried out using the Multcomp package for R[^1^](https://paperpile.com/c/qB23uq/V0Dp), performing Wald tests on a global 5% significance level to test the response of different dilutions vs. control within every time point.

A bead attraction assay was carried out to validate the attraction of MT+ to diproline after SIP- treatment. Oasis® solid phase Extraction cartridges (Waters Corporation) were equilibrated with 1 ml methanol and washed two times in MQ water, before loading 2 ml 0.00514 M synthetic diproline. Afterwards, the silica beads inside the cartridges were eluted in 2 ml MQ and subsequently diluted 1/50. Exponentially growing cultures of MT+ (strain 85A) were grown in a 24-well plate. After dark-synchronization of the cultures in G1-phase 50 µl of beads was added. Four different treatments were tested: (1) control, no conditioning with SIP-, no diproline on the beads; (2) conditioning with SIP-, no diproline; (3) no conditioning with SIP-, diproline on the beads; and (4) both conditioned with SIP and diproline loaded beads. For every well, a microscopic picture was taken from three random beads, 10 min after adding the beads. This procedure was repeated at 0.25h, 1h, 3h, 6h and 9h. Attraction was quantified by counting the number of cells in a 50 µm radius around the bead using image manipulation software ImageJ. For statistical analysis, we adopted a generalized linear mixed-effects model with Poisson distribution and log link function using the lme4 package for R (version 1.1-18-1[^2^](https://paperpile.com/c/qB23uq/9qBJO)). The number of cells was modeled as a function of treatment, time and their interaction effects as fixed effects. The effects of replicate and pseudoreplicate were added as random effects to account for multiple wells assessed for every replicate. Pairwise comparisons were carried out using Wald tests implemented in the glht function from the multcomp package for R. Attraction of beads was compared within every time point between each treatment and the control.

# Cell cycle analysis using flow cytometry

Cultures of MT+ (strain 85A) and MT- (strain PONTON34) were subjected to a 36h dark arrest to synchronize them in the G1 phase of the cell cycle[^3^](https://paperpile.com/c/qB23uq/OBhue). Before re-illumination, half of the cultures were treated with a 1/10x dilution of SIP filtrate from the other mating type. The same batch of SIP- filtrate was used for all experimental repeats, and was kept at 4°C in between repeats. Subsequently, 10 ml of culture was harvested at set time points (1h, 3h, 6h and 9h) and samples were centrifuged for 5 min at 1000 RPM. The supernatant was discarded and 10 ml ice cold 75% ethanol was added for fixation. The pellet was resuspended and stored in the dark at 4°C until further analysis. Later, fixed cultures were centrifuged for 5 min at 3000 RPM, after which the supernatant was replaced with 2 ml ice cold 75% ethanol. One ml of each sample was transferred to a 1.5 ml tube which was washed three times with PBS buffer. The fixed cells were treated with 1 µg/ml RNAse A for 20 min at 37°C, after which MT+ and MT- cultures were stained with propidium iodide (PI, 50µg/ml) and SYBR green (concentration 1x) respectively. Samples were filtered through a cell strainer with pore size of 70 µm and then analyzed on a Bio-Rad S3e cell sorter (BioRad laboratories, inc.). Data analysis was performed using the FlowCore 1.44.2[^4^](https://paperpile.com/c/qB23uq/3wSKJ) and ggcyto 1.9.12[^5^](https://paperpile.com/c/qB23uq/QQMoI) packages for R. Debris was removed by gating and filtering on the forward scatter (FSC) versus side scatter (SSC) scatterplot, followed by a second gating step where unstained particles were removed. The number of cells in G1 and G2/M was identified by gating on the midpoint between both peaks on the histogram of intensities in the FL3 or FL1 channel respectively for MT+ and MT-. Statistical inference was carried out based on a quasibinomial generalized linear model (GLM) to account for overdispersion. The fraction of dividing cells was modelled as a function of treatment, time and their interaction effect. We accounted for the blocking structure in the experimental design of the MT+ experiment with a fixed effect for replicate. A global Wald test was performed as an omnibus test to assess any effect of the treatment in every time point. Upon rejection of the global test, a post-hoc analysis is carried out using Wald tests implemented in the multcomp R package[^1^](https://paperpile.com/c/qB23uq/V0Dp) on a global 5% significance level, testing the response of treatment vs. control on the proportion of G2 cells within every time point.

# Assessing the effect of SIP- on MT+ motility

*S. robusta* MT+ (strain 85A) was grown at 18 °C under 20-25 µmol m^-2^s^-1^ fluorescent white light. Exponentially growing cells were inoculated in a 24-well multiwell plate (CELLSTAR®, Greiner Bio-one) filled with 1 ml of medium. The resulting cell density was 42.5 ± 16.6 cells mm^-2^ (± SD) and cell length was 30.0 ± 1.8 µm (± SD, n = 30). At the end of the 36h dark-synchronisation period, 1 ml of SIP- filtrate was added to achieve a final dilution of 1/10x the original concentration. A control without the pheromone was included by adding medium without SIP. Immediately afterwards, the cultures were transferred to the light. For each well, a 30-second microscopic movie was recorded every hour over the course of 9 hours. Quantification of motility over the 30 seconds period was performed with a custom MATLAB script (Suppl. Fig. 11).

Every movie consisted of 11 frames and was exported as a multi-page tiff file. Every frame was imported in MATLAB as a gray-scale image and converted to a binary image. The centroids of the cells were used as cell coordinates and linked to a cell path by a MATLAB adaptation of John C. Crocker’s particle tracking code[^6^](https://paperpile.com/c/qB23uq/NbGqt). Prior to the analysis, the dark corners of the image were corrected. For the analysis of the frames, the frames were filtered using a LoG-filter that acts as an edge detection filter. Removal of the generated noise and cell clusters yields a binary image consisting of single cells only. The output of the tracking code was a set of coordinates belonging to a path for every cell. The total length of the path was calculated and was used to determine if a cell was moving or not using a threshold-based procedure: since non-moving cells also generated a small path length, a set of movies without moving cells was generated by manually removing moving cells, and fed into the same algorithm described above. The resulting data was used to calculate a threshold for moving cells. The threshold was calculated to be the 95% quantile of the generated path lengths under no motility. Cells with a path length lower than the generated threshold were defined to be non-moving, those with a path length greater than the threshold were set to moving. For statistical inference we fitted a quasibinomial GLM to account for overdispersion. The expected fraction of motile cells was modeled as a function of treatment, time and their interaction effect. A global Wald test was performed to assess any effect of the treatment at every time point. Upon rejection of the global test, pairwise comparisons using Wald tests implemented in the glht function from the multcomp package for R[^1^](https://paperpile.com/c/qB23uq/V0Dp). The fraction of motile cells was compared within every time point between the 4 levels of dilution and the control. An extended Figure including multiple dilution levels of the SIP- filtrate is included as Suppl. Fig. 12.

# MT+ RNA-seq dataset experimental setup, RNA extraction, sequencing and processing

MT+ cells (strain 85A) were grown in 150 mL Cellstar® culture flasks (Greiner Bio-One GmbH, Austria). Two days before harvesting, the medium was replaced with fresh NSW + F/2 + antibiotics, and the density of the cultures was assessed using the F0 metric on a Maxi-PAM (Walz GmbH, Germany)[^7^](https://paperpile.com/c/qB23uq/wHB3) using parameters Intensity 7, Gain 3 and Damping 2. Cultures were subsequently diluted to an F0 value of 0.1. Before the start of the experiment, cultures were kept in the dark for 36h to ensure cell cycle synchronization in the G1 phase. Before re-illumination, half of the cultures in 150 mL of medium were treated with 15 mL of SIP- filtrate. Fifteen minutes after addition of the filtrate, the light was turned on. At each of five time points (15min, 1h, 3h, 6h and 9h), two technical replicate flasks of SIP- treated and untreated cultures were harvested by filtration on a Versapor® filter with a pore size of 3 µm (Pall Corporation, NY, USA). The filters were subsequently rinsed with 1 mL PBS buffer, flash-frozen in liquid nitrogen and kept at -80°C until RNA extraction. The experiment was repeated on 3 different occasions to obtain 3 independent replicates.

Cells were scraped from the filters and lysed by adding 1 ml RLT lysis buffer containing 10µl β-mercaptoethanol. Silicon carbide beads (1 mm, Bio spec products Inc.) were added and the tubes were put in a beating mill (Retsch GmbH) for 30 minutes at 20 Hz. To extract RNA from the lysate, an RNeasy plant mini kit (Qiagen) was used according to the manufacturer’s instructions, including an on-column DNAse treatment. One µl of each replicate was analyzed using a BioAnalyzer pico chip (Agilent technologies) to ensure RNA quality and concentration. The technical replicate of each repeat with best quality (BioAnalyzer RIN-value) and quantity (RNA concentration) was selected for sequencing. Library preparation and 2x75bp paired-end sequencing on the Illumina NextSeq500 platform occurred at VIB Nucleomics core (Leuven, Belgium <http://www.nucleomics.be/>). An average of 26.7±2.6 million reads was sequenced per sample. The samples were divided over two runs according to a randomized block design: all samples from the first repeat were assigned to the first run, and all samples of the second repeat were assigned to the second run, while samples of the third repeat were divided over the two runs in such a way that different treatments of one time point were within the same run. This block design allows an unbiased comparison of treatment effects, since all contrasts of interest can be assessed within a single run.

RNA-seq data generated in this study representing the response of MT+ to SIP- was complemented with existing data on the response of MT- to SIP+. We retrieved raw paired-end reads from Moeys et al. (2016)[^8^](https://paperpile.com/c/qB23uq/uCUqx) for time points 15min, 1h and 3h, and from Cirri et al. (2019)[^9^](https://paperpile.com/c/qB23uq/loh0U) for a time point of 10h after re-illumination. In total, 54 samples were analyzed: 30 MT+ samples comprising 5 time points and 24 MT- samples comprising 4 time points. Quality control was performed on all samples using FastQC (Babraham Bioinformatics, under GPL3 license). Reads from Moeys et al. (2016) contained adaptor sequences which were trimmed with cutadapt v1.8[^10^](https://paperpile.com/c/qB23uq/Vd3yS). All three RNA-seq datasets were mapped to gene models from the *S. robusta* genome v1.0 (available at <https://bioinformatics.psb.ugent.be/orcae/overview/Semro>) with Salmon v0.9.1. The average mapping rate was 77.7±5.7% for MT+, 66.9±13.9% for MT- from Moeys et al (2016)[^8^](https://paperpile.com/c/qB23uq/uCUqx) and 83.1±1.2% for MT- generated by Cirri et al (2019)[^9^](https://paperpile.com/c/qB23uq/loh0U). Note that the mapping rate is higher in the newer RNA-seq datasets as a result of improved data quality (Suppl. Fig. 1A).

Separate differential expression (DE) analyses were performed for each of the three datasets (i.e., the new MT+ RNA-seq time-series and two existing MT- RNA-seq datasets[^8,9^](https://paperpile.com/c/qB23uq/loh0U+uCUqx)). Isoform-level abundances were imported in R using the tximport package (v1.8.0)[^11^](https://paperpile.com/c/qB23uq/bjDBo) and aggregated to the gene level. Independent filtering[^12^](https://paperpile.com/c/qB23uq/GILdp) was performed by retaining genes with at least 1 count per million (CPM) in at least three samples. Differences in sequencing depth and RNA population were corrected for by adopting TMM normalization[^12,13^](https://paperpile.com/c/qB23uq/GILdp+idvNr) and including the natural logarithm of effective library sizes as offsets to the model. Negative binomial GLMs were estimated for every gene with edgeR[^14^](https://paperpile.com/c/qB23uq/NhIgr), and gene expression was modelled as a function of an interaction between treatment and time. For the novel MT+ data and the MT- data from Moeys et al (2016) we account for technical effects by incorporating main effects of run and replicate, to respectively account for the sequencing run effects and block design imposed by the data generation structure (i.e., the data for different replicates may be gathered in different weeks). DE between treatment and control at every time point was tested for using likelihood ratio tests (LRT). Since interpretation and biological validation occur at the gene level, we adopted stage-wise testing to control the gene-level false discovery rate (FDR)[^15^](https://paperpile.com/c/qB23uq/v0XnM).

# Functional annotation of *Seminavis robusta* genes

Functional annotation for all *S. robusta* genes was derived using three different approaches: (i) InterProScan v5.3[^16^](https://paperpile.com/c/qB23uq/rcrnN) was executed to search for matches against the InterPro protein signature databases; (ii) AnnoMine[^17^](https://paperpile.com/c/qB23uq/MQxAf) was applied for consensus gene functional annotation retrieval from protein similarity searches (using DIAMOND v0.9.9.110[^18^](https://paperpile.com/c/qB23uq/G0ntn), maximum e-value 10e-05) against the Swiss-Prot database[^19^](https://paperpile.com/c/qB23uq/jhm7S); (iii) eggNOG-mapper[^20^](https://paperpile.com/c/qB23uq/43svR) was used in DIAMOND mapping mode, based on eggNOG 4.5 orthology data[^21^](https://paperpile.com/c/qB23uq/fwVBk). To compute gene families, an all-against-all protein similarity search was performed using DIAMOND (maximum e-value 10e-05, max 4000 hits), after which the protein sequences were clustered into families using TRIBE-MCL v10-201[^22^](https://paperpile.com/c/qB23uq/I9Q6C). Functional annotation and gene family information for 10 diatom species can be found on the diatom-PLAZA platform for comparative genomics (https://bioinformatics.psb.ugent.be/plaza/versions/plaza_diatoms_01/).

The presence of specific conserved motifs was confirmed using multiple sequence alignment in CLC Main Workbench (Qiagen) for GDPH-domain containing proteins[^23^](https://paperpile.com/c/qB23uq/opfU), frustulins[^24^](https://paperpile.com/c/qB23uq/3MekM), sin1-homologues[^25^](https://paperpile.com/c/qB23uq/iz1PC), silicic acid transporters and guanylate cyclases (GC)[^26^](https://paperpile.com/c/qB23uq/MnTJS). To verify the specificity for guanine over adenine, the amino acid sequence of the catalytic domain of GCs was verified using multiple sequence alignment with MEGA7[^26^](https://paperpile.com/c/qB23uq/MnTJS) and the presence of transmembrane domains was determined using Phobius[^27^](https://paperpile.com/c/qB23uq/AnDr). To characterize the cyclin family in *S. robusta*, a phylogenetic analysis was performed using 56 putative S. robusta cyclins and a selection of cyclins from *P. tricornutum*, *T. pseudonana* and *A. thaliana*. The *A. thaliana* SDS (At1g14750) amino acid sequence was used as an outgroup. Multiple sequence alignment was carried out with MAFFT v7.187[^28^](https://paperpile.com/c/qB23uq/sIJF) after which poorly aligned positions were trimmed using Trimal V1.4.1 (-gt 0.1)[^29^](https://paperpile.com/c/qB23uq/vW1y). The phylogenetic tree was constructed using IQ-TREE v1.7 (-bb 1000 -mset JTT,LG,WAG,Blosum62,VT,Dayhoff -mfreq F -mrate R)[^30^](https://paperpile.com/c/qB23uq/dspH).

To identify known sex induced genes in the genome of *Seminavis robusta*, BLASTp searches were carried out based on amino acid sequences from *Skeletonema marinoi* and *Pseudo-nitzschia multistriata*. Afterwards, amino acid sequences from each species were used for InterPro domain prediction[^31^](https://paperpile.com/c/qB23uq/yeXU).

# Gene set enrichment analysis

Gene ontology (GO) enrichment analyses were adopted to interpret transcriptional changes observed at every time point for each mating type. Competitive gene set testing was carried out on GO terms predicted by the EggNOG-mapper with CAMERA[^32^](https://paperpile.com/c/qB23uq/1yxyk). Briefly, CAMERA tests whether a set of genes is highly ranked for the hypothesis of interest as compared to other genes outside the gene set, while accounting for inter-gene correlation. Using CAMERA, a set of GO terms was defined for every time point of every mating type that are enriched in significant genes at the 5% FDR level. Enriched GO terms were summarized using REVIGO[^33^](https://paperpile.com/c/qB23uq/ASPpY) using a similarity cut-off value of 0.5 and the SimRel score as a similarity measure[^34^](https://paperpile.com/c/qB23uq/s9pX). REVIGO-summarized terms involved in processes of interest were selected and their enrichment was verified in other time points from both mating types. We selected GO terms from processes of interest and results were plotted in a heatmap showing the enrichment of each term in each time point and mating type combination (Suppl. Fig. 2).

# Integrative analysis

SRBs, i.e. genes with a response to SIP in both mating types, were selected by testing whether their fold change was significantly higher than 3 or lower than 1⁄3 in at least one time point using a test for DE relative to a fold change threshold[^8,35^](https://paperpile.com/c/qB23uq/uCUqx+2MZHX). To control the gene-level false discovery rate (FDR) on a 5% level for each dataset, a stage-wise testing approach was used[^15^](https://paperpile.com/c/qB23uq/v0XnM) based on Sidak p-value aggregation. We first select these genes in the MT- dataset, and subsequently analyze this subset in the MT+ dataset using the same approach, i.e., we test against the same fold change threshold in at least one time point on a 5% gene-level FDR for the subset of MT- significant genes. This sequential analysis allows for a lower burden on the multiple testing correction in the novel MT+ dataset. Note that this workflow focusses the FDR control on the final step of the analysis.

In contrast to SRBs, a different two-step procedure involving equivalence testing was used to discover genes that respond to the pheromone in only one mating type (SRMs and SRPs), see Suppl. Table 5 below. In a first step non-responsive genes were detected for each mating type through equivalence testing[^36^](https://paperpile.com/c/qB23uq/f3YUZ) by selecting genes whose fold change was significantly contained within an equivalence interval ranging from ⅓ to 3 on a 5% FDR level. The equivalence tests were performed using the two one-sided tests (TOST) procedure for every contrast. To test for genes that are equivalent across all 3 time points, we controlled the FDR on the maximum p-value across all time points for a given gene. Since genes that are not expressed in a particular mating type (MT) can also be considered to be non-responsive, equivalent genes were subsequently merged with lowly expressed genes (CPM < 1 in > 3 samples) that were previously filtered out of the analysis. Next, we set out to identify which of the non-responsive genes in one MT were responsive in the compatible mating type. We identified genes with a fold change significantly higher than 3 or lower than ⅓ in at least one time point using an omnibus test on a 5% FDR level, producing lists of genes that are responsive in one MT, but non-responsive in the other. Expression in control and SIP treated conditions was plotted for a selection of candidate genes from each class (Fig. 3).

**Supplementary Table 5**: Key genes as defined by their behavior in response to treatment with SIP. SRP and SRM genes are identified using a combination of equivalence testing and differential expression testing, while SRB genes are identified using differential expression testing in two separate datasets.

|  | Expression in MT+ | Expression in MT- |
| --- | --- | --- |
| SRP | DE, \|log fold change\| > log(3) | Not responsive (equivalent / low expression) |
| SRM | Not responsive (equivalent / low expression) | DE, \|log fold change\| > log(3) |
| SRB | DE, \|log fold change\| > log(3) | DE, \|log fold change\| > log(3) |

# Visualisation of genes of interest

We investigated the response over the entire time interval of genes of interest using heatmaps which show the expression of significant genes in each time point for each mating type. Average counts per million (CPM) were calculated for every treatment*time combination. To improve visualization, average CPM were scaled separately for each mating type to zero mean and unit variance within each gene, except for supplementary heatmaps showing a mating type specific response, where scaling was performed over both mating types for each gene. All heatmaps were plotted with the ggplot2 package[^37^](https://paperpile.com/c/qB23uq/shDzC) using a gradient palette from the R package ‘wesanderson'[^38^](https://paperpile.com/c/qB23uq/5jsKc). Heatmaps show CPM relative to the overall mean expression: expression was standardized to zero mean and unit variance for each gene. To explore the synchronized expression of cyclins in the control samples during the time series, cyclins with changing expression in both mating types over time were selected (maximum CPM must exceed 2 times the minimum CPM) and ordered based on their expression profile using hierarchical clustering in R.

# Strain usage across experiments

Due to size decline of strains as well as issues with the growth and health of mother cultures, different strains have been used to represent MT+ and MT-. For most experiments concerning MT+ strain 85A was used, except for SIP+ production (strain D6). For most MT- experiments, we used strain PONTON34, except for SIP- filtrate production (strain 85A); the latter is the strain used by Moeys and colleagues for transcriptomics analysis of MT-[^8^](https://paperpile.com/c/qB23uq/uCUqx). All strains belong to *S. robusta* Clade I, which displays high fecundity in pairwise crosses within the clade, demonstrating that pheromone signaling and mating partner recognition is conserved between strains of this clade[^39^](https://paperpile.com/c/qB23uq/VVmE). Choice of strain for all experiments discussed in this work is summarized in Suppl. Table 1.

# Rendering cultures for RNA-seq axenic

Prior to RNA-seq experiments, cultures were treated with medium containing a mix of 500 mg/L penicillin, 500 mg/L ampicillin, 100 mg/L streptomycin and 50 mg/L gentamycin for one week before the experiment. These concentrations have been tested and were successful in rendering diatom cultures axenic in the past[^40^](https://paperpile.com/c/1EUmH8/VbTx).

# References

1. [Hothorn T, Bretz F, Westfall P. Simultaneous inference in general parametric models. Biometrical Journal. 2008;50:346–63.](http://paperpile.com/b/qB23uq/V0Dp)

2. [Bates D, Mächler M, Bolker B, Walker S. Fitting linear mixed-effects models using lme4. Journal of Statistical Software. 2014;67:2015.](http://paperpile.com/b/qB23uq/9qBJO)

3. [Gillard J, Devos V, Huysman MJJ, De Veylder L, D’Hondt S, Martens C, et al. Physiological and transcriptomic evidence for a close coupling between chloroplast ontogeny and cell cycle progression in the pennate diatom *Seminavis robusta*. Plant Physiol. 2008;148:1394–411.](http://paperpile.com/b/qB23uq/OBhue)

4. [Hahne F, LeMeur N, Brinkman RR, Ellis B, Haaland P, Sarkar D, et al. flowCore: a Bioconductor package for high throughput flow cytometry. BMC Bioinformatics. 2009](http://paperpile.com/b/qB23uq/3wSKJ);10:106:

5. [Van P, Jiang W, Gottardo R, Finak G. ggCyto: next generation open-source visualization software for cytometry. Bioinformatics. 2018;34:3951–3.](http://paperpile.com/b/qB23uq/QQMoI)

6. [Blair, D.; Dufresne, E. The matlab marticle tracking code repository. Department of Physics, Georgetown University: Washington, DC, 2010;](about:blank) <http://www.physics.georgetown.edu/matlab/index.html>. V[isited 2019 Aug 20](http://paperpile.com/b/qB23uq/NbGqt)

7. [Stock W, Blommaert L, Daveloose I, Vyverman W, Sabbe K. Assessing the suitability of imaging-PAM fluorometry for monitoring growth of benthic diatoms. Journal of Experimental Marine Biology and Ecology. 2019;513:35–41.](http://paperpile.com/b/qB23uq/wHB3)

8. [Moeys S, Frenkel J, Lembke C, Gillard JTF, Devos V, Van den Berge K, et al. A sex-inducing pheromone triggers cell cycle arrest and mate attraction in the diatom *Seminavis robusta*. Sci Rep. 2016;6:19252.](http://paperpile.com/b/qB23uq/uCUqx)

9. [Cirri E, De Decker S, Bilcke G, Werner M, Osuna-Cruz CM, De Veylder L, et al. Associated bacteria affect sexual reproduction by altering gene expression and metabolic processes in a biofilm inhabiting diatom. Front Microbiol. 2019;10:1790.](http://paperpile.com/b/qB23uq/loh0U)

10. [Martin M. Cutadapt removes adapter sequences from high-throughput sequencing reads. EMBnet journal. 2011;17:10.](http://paperpile.com/b/qB23uq/Vd3yS)

11. [Soneson C, Love MI, Robinson MD. Differential analyses for RNA-seq: transcript-level estimates improve gene-level inferences. F1000Res. 2015;4:1521.](http://paperpile.com/b/qB23uq/bjDBo)

12. [Bourgon R, Gentleman R, Huber W. Independent filtering increases detection power for high-throughput experiments. Proc Natl Acad Sci U S A. 2010;107:9546–51.](http://paperpile.com/b/qB23uq/GILdp)

13. [Robinson MD, Oshlack A. A scaling normalization method for differential expression analysis of RNA-seq data. Genome Biol. 2010;11:R25.](http://paperpile.com/b/qB23uq/idvNr)

14. [McCarthy DJ, Chen Y, Smyth GK. Differential expression analysis of multifactor RNA-Seq experiments with respect to biological variation. Nucleic Acids Res. 2012;40:4288–97.](http://paperpile.com/b/qB23uq/NhIgr)

15. [Van den Berge K, Soneson C, Robinson MD, Clement L. stageR: a general stage-wise method for controlling the gene-level false discovery rate in differential expression and differential transcript usage. Genome Biol. 2017;18:151.](http://paperpile.com/b/qB23uq/v0XnM)

16. [Jones P, Binns D, -Y. Chang H, Fraser M, Li W, McAnulla C, et al. InterProScan 5: genome-scale protein function classification. Bioinformatics. 2014;30:1236–40.](http://paperpile.com/b/qB23uq/rcrnN)

17. [Vandepoele K, Van Bel M, Richard G, Van Landeghem S, Verhelst B, Moreau H, et al. pico-PLAZA, a genome database of microbial photosynthetic eukaryotes. Environ Microbiol. 2013;15:2147–53.](http://paperpile.com/b/qB23uq/MQxAf)

18. [Buchfink B, Xie C, Huson DH. Fast and sensitive protein alignment using DIAMOND. Nat Methods. 2014;12:59–60.](http://paperpile.com/b/qB23uq/G0ntn)

19. [Bairoch A, Apweiler R. The SWISS-PROT protein sequence database and its supplement TrEMBL in 2000. Nucleic Acids Res. 2000;28:45–8.](http://paperpile.com/b/qB23uq/jhm7S)

20. [Huerta-Cepas J, Forslund K, Coelho LP, Szklarczyk D, Jensen LJ, von Mering C, et al. Fast genome-wide functional annotation through orthology assignment by eggNOG-mapper. Mol Biol Evol. 2017;34:2115–22.](http://paperpile.com/b/qB23uq/43svR)

21. [Huerta-Cepas J, Szklarczyk D, Forslund K, Cook H, Heller D, Walter MC, et al. eggNOG 4.5: a hierarchical orthology framework with improved functional annotations for eukaryotic, prokaryotic and viral sequences. Nucleic Acids Res. 2016;44:286–93.](http://paperpile.com/b/qB23uq/fwVBk)

22. [Enright AJ, Van Dongen S, Ouzounis CA. An efficient algorithm for large-scale detection of protein families. Nucleic Acids Res. 2002;30:1575–84.](http://paperpile.com/b/qB23uq/I9Q6C)

23. [Lachnit M, Buhmann MT, Klemm J, Kröger N, Poulsen N. Identification of proteins in the adhesive trails of the diatom *Amphora coffeaeformis*. Philos Trans R Soc Lond B Biol Sci. 2019;374:20190196.](http://paperpile.com/b/qB23uq/opfU)

24. [Kroger N, Bergsdorf C, Sumper M. Frustulins: Domain conservation in a protein family associated with diatom cell walls. Eur J Biochem. 1996;239:259–64.](http://paperpile.com/b/qB23uq/3MekM)

25. [Kotzsch A, Gröger P, Pawolski D, Bomans PHH, Sommerdijk NAJM, Schlierf M, et al. Silicanin-1 is a conserved diatom membrane protein involved in silica biomineralization. BMC Biol. 2017;15:65.](http://paperpile.com/b/qB23uq/iz1PC)

26. [Winger JA, Derbyshire ER, Lamers MH, Marletta MA, Kuriyan J. The crystal structure of the catalytic domain of a eukaryotic guanylate cyclase. BMC Struct Biol. 2008;8:42.](http://paperpile.com/b/qB23uq/MnTJS)

27. [Käll L, Krogh A, Sonnhammer ELL. A combined transmembrane topology and signal peptide prediction method. Journal of Molecular Biology. 2004;338:1027–36.](http://paperpile.com/b/qB23uq/AnDr)

28. [Katoh K, Standley DM. MAFFT multiple sequence alignment software version 7: improvements in performance and usability. Molecular Biology and Evolution. 2013;30:772–80.](http://paperpile.com/b/qB23uq/sIJF)

29. [Capella-Gutiérrez S, Silla-Martínez JM, Gabaldón T. TrimAl: a tool for automated alignment trimming in large-scale phylogenetic analyses. Bioinformatics. 2009;25:1972–3.](http://paperpile.com/b/qB23uq/vW1y)

30. [Nguyen L-T, Schmidt HA, von Haeseler A, Minh BQ. IQ-TREE: a fast and effective stochastic algorithm for estimating maximum-likelihood phylogenies. Mol Biol Evol. 2015;32:268–74.](http://paperpile.com/b/qB23uq/dspH)

31. [Mitchell AL, Attwood TK, Babbitt PC, Blum M, Bork P, Bridge A, et al. InterPro in 2019: improving coverage, classification and access to protein sequence annotations. Nucleic Acids Res. 2019;47:351–60.](http://paperpile.com/b/qB23uq/yeXU)

32. [Wu D, Smyth GK. Camera: a competitive gene set test accounting for inter-gene correlation. Nucleic Acids Res. 2012;40:e133.](http://paperpile.com/b/qB23uq/1yxyk)

33. [Supek F, Bošnjak M, Škunca N, Šmuc T. REVIGO summarizes and visualizes long lists of gene ontology terms. PLoS One. 2011;6:e21800.](http://paperpile.com/b/qB23uq/ASPpY)

34. [Schlicker A, Domingues FS, Rahnenführer J, Lengauer T. A new measure for functional similarity of gene products based on Gene Ontology, BMC Bioinformatics. 2006;7:302.](http://paperpile.com/b/qB23uq/s9pX)

35. [McCarthy DJ, Smyth GK. Testing significance relative to a fold-change threshold is a TREAT. Bioinformatics. 2009;25:765–71.](http://paperpile.com/b/qB23uq/2MZHX)

36. [Derycke S, Kéver L, Herten K, Van den Berge K, Van Steenberge M, Van Houdt J, et al. Neurogenomic profiling reveals distinct gene expression profiles between brain parts that are consistent in *Ophthalmotilapia* cichlids. Front Neurosci. 2018;12:136.](http://paperpile.com/b/qB23uq/f3YUZ)

37. [Wickham H. ggplot2: Elegant graphics for data analysis. Springer; 2016:260.](http://paperpile.com/b/qB23uq/shDzC)

38. [Ram K, Wickham H. wesanderson: A Wes Anderson palette generator. 2018. Available from:](http://paperpile.com/b/qB23uq/5jsKc) <https://github.com/karthik/wesanderson>

39. [De Decker S, Vanormelingen P, Pinseel E, Sefbom J, Audoor S, Sabbe K, et al. Incomplete reproductive isolation between genetically distinct sympatric clades of the pennate model diatom *Seminavis robusta*. Protist. 2018;169:569–83.](http://paperpile.com/b/qB23uq/VVmE)

40. [Koedooder C, Stock W, Willems A, Mangelinckx S, De Troch M, Vyverman W, et al. Diatom-bacteria interactions modulate the composition and productivity of benthic diatom biofilms. Front Microbiol. 2019;10:1255.](http://paperpile.com/b/1EUmH8/VbTx)
