# Supplementary figures and images for: Mating type specific transcriptomic response to sex inducing pheromone in the pennate diatom Seminavis robusta
Source: ISME J. 2020 Oct 7;15(2):562–76. doi: 10.1038/s41396-020-00797-7 (PMC8027222; doi:10.1038/s41396-020-00797-7)

# SRBs

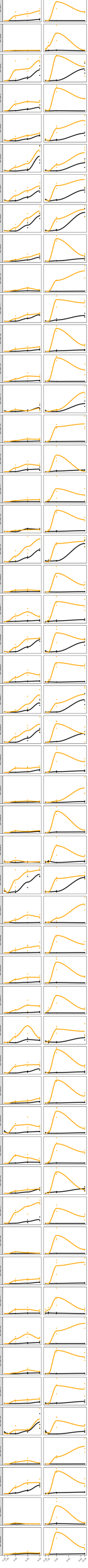

Supplement: Supplementary file 3 — Supplementary Figure 3 [file 41396_2020_797_MOESM3_ESM.pdf]

## SRMs

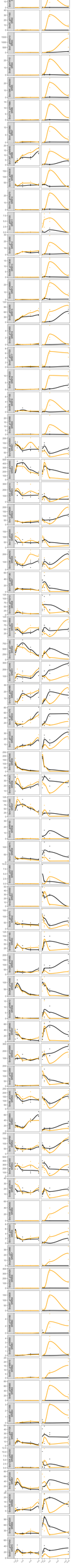

Supplement: Supplementary file 4 — Supplementary Figure 4 [file 41396_2020_797_MOESM4_ESM.pdf]

# SRPs

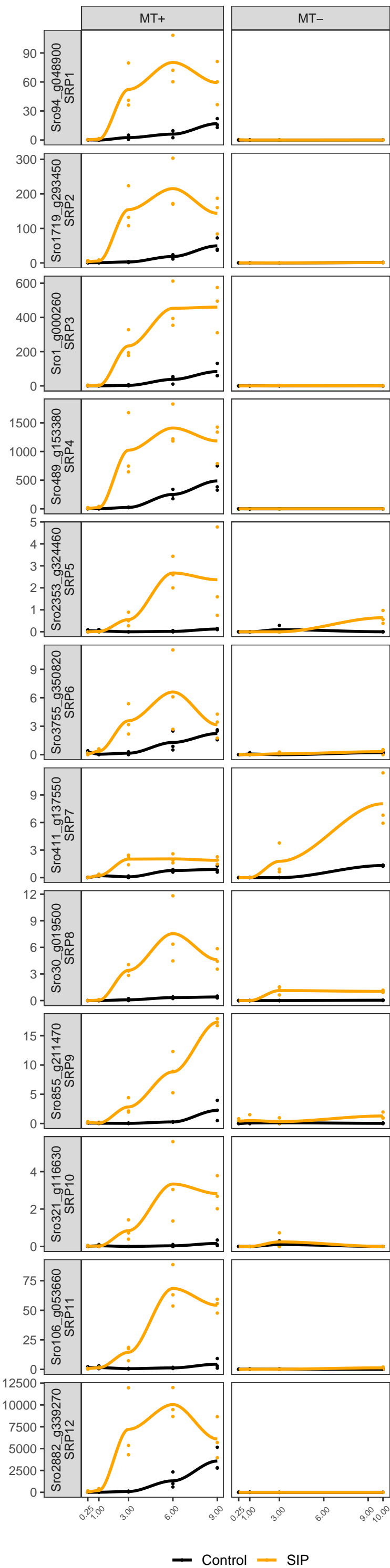

Supplement: Supplementary file 5 — Supplementary Figure 5 [file 41396_2020_797_MOESM5_ESM.pdf]
